# Medico-Chirurgical Notes and Illustrations. Part I.

**Published:** 1831

**Authors:** 


**MEDICO-CHIRURGICAL NOTES AND ILLUSTRATIONS. PART I. ON SOME DANGEROUS AFFECTIONS OF THE THROAT WHICH INDUCE SUDDEN DEATH BY SUFFOCATION. ON STRICTURES OF THE ŒSOPHAGUS, AND THE DANGERS OF THE BOUGIE. ON THE CURE OF THE FALLING DOWN OF THE BOWEL IN GROWN PERSONS. ANOMALIES IN RUPTURE OPERATIONS, &c. &c. &c. By *R. Fletcher*, Esq. Surgeon to the General Infirmary at Gloucester, &c. 4to, pp. 146. Four lithog. Plates. Longman's, London, 1831.**

THERE are two very opposite descriptions of writers and writings in medical science, and each are of value in their way. We have plain and practical authors with little pretension to theory or principle, and but slight powers of inductive or deductive generalization; and we have men of more speculative talent, who delight in deducing laws from facts, and who have ever been the founders of our theoretical systems. It would not be difficult to prove that if either party were exclusively predominant, the world would be the loser. If facts were deficient, theories would be worse than they are; and if theories were abolished, facts would be of comparatively little service. To expect in the generality of men a happy combination of patience in observing facts with consummate ability, in drawing conclusions from them, is to look for that which the wise Author of Nature has determined should not be. It is amusing to observe the feud which rages and has ever raged between the two classes of persons to whom we have alluded. From the dawn of science they have ever been at variance, and have ever striven for rule and mastery. Yet we find them in much the same position now, as they occupied some forty centuries ago; a plain proof to our minds, since every thing is ordered for the best, that they always will hold the same relative situation, and that the interests of mankind require that they should do so.

The author of these "Notes and Illustrations" is of the practical school, an empiric rather than a dogmatist. He tells us that "he has no other to offer than the truth." If he gives us so much we shall feel well satisfied, for in these miraculous days of intelligence truth has grown somewhat too scarce. Perchance she is Tory in her notions, and will not march with the intellectual regiment of sans culottes.

The present work, then, is not ushered in with high pretensions, but to practical surgeons the observations which it contains will be useful, and to all they will be interesting. Mr. Fletcher apologizes for the colloquial manner in which they are related, and hints that some domestic affliction has induced him to give them to the public as they are. We shall put our readers in possession of what is valuable in the volume, and we doubt not that Mr. Fletcher will derive satisfaction from knowing that his reflections in the chambers of the dying and the dead, are borne by the mercurial speed of the periodical press to every corner of the old world and the new. As we have lately devoted some space to the consideration of "calculous disorders," we shall commence the present article with a chapter on failures in lithotomy, although it is by no means the first in the work.

### On Failures in Lithotomy.

It is an excellent plan, as Mr. F. observes, for a surgeon, on losing a patient after operation, to imagine that something in that operation was amiss. If he does not do so, if he be of the temper of Candide, and lay the flattering unction to his soul that he did what man could do, and that his patient died simply because he could not live, in all probability that surgeon will never improve. After every unsuccessful operation we must first imagine that some step was wrong, and secondly, we must endeavour to determine where the error lay, and how it may hereafter be avoided. This philosophic rule is especially valuable when applied to lithotomy, for so much in its performance is done in the dark, that frequently the operator alone can determine in what he has erred, and even he will be misled unless he exercise a careful analysis and induction.

The invention of lithotrity and the introduction of the forceps for extracting small calculi will undoubtedly constitute an era in surgery, and although we do not imagine that lithotomy will be altogether superseded, yet we do believe that when these two improvements are properly appreciated and their consequences fairly followed out, we do believe, we say, that when this is accomplished, lithotomy will become much less frequent than it is. This need not prevent us from endeavouring to ascertain, as far as that is possible, the reasons for the acknowledged fatality of the operation; on the contrary, it is imperative upon us to endeavour to learn what can be learnt upon the subject, if practical experience is to be more scarce. We hailed with pleasure the appearance of Mr. Brodie's Lectures on Calculous Disorders, and to our analysis of those lectures in the last number of this Journal we refer, for that distinguished surgeon's views of the causes of death from lithotomy. Our readers then will find, or will recollect, if they have read the article to which we allude, that Mr. Brodie considers peritonitis as a much less frequent cause of death than it is commonly considered to be. His observations and dissections have led him to the opinion, that fatal inflammation and sloughing of the cellular membrane of the pelvis, giving rise, it is true, to peritonitis towards the close, is infinitely more common. We shall see how far the conclusions drawn by Mr. Fletcher tally with those of Mr. Brodie. With the following sentiment we perfectly agree, but alas! how Utopian to conceive that men will publish their failures. Why, nine out of ten have not even the honesty to attach only the proper degree of merit and value to their success!

"If hospital surgeons, who do this operation most frequently, were to report the failures they have witnessed, and the circumstances which attended them, much might be done towards abbreviating the sufferings, and preserving the lives of patients. The history of failures is, perhaps, more valuable than that of successful cases. Dissection will trace the causes of death, with the errors committed, and point out how, in future, they can be avoided, so as to lead to more precision, and certainty of a successful termination. A broken down sufferer of many years, who has made up his mind to submit to a terrible operation, the climax of pain and punishment, relying on our skill, judgment, and humanity, for its being safely done, should be considered as a patient of the whole profession. He has placed life, his last and most precious stake in its hands; and every member, whose experience allows him an opportunity, should not hesitate to contribute his mite to its preservation, by recounting, as warnings, the failures that he has beheld." 81.

Mr. Fletcher starts with deprecating a race against time in the performance of the operation. He might have saved himself that trouble. If men imagine that they can operate with rapidity and dexterity, they will endeavour to do so to the exclusion of some other considerations, so long, at least, as vanity shall continue a component of human nature. The next error against which Mr. Fletcher directs his censure is the employment of violence.

"It will be found that their main source is violence, generally, though not always from the forceps, on whose blade should be engraven the motto, 'Gardez bien.' This violence is often unnecessary, for it is better to cut, than to bruise and lacerate, in the extraction of large calculi; to cut the bladder again and again, than to tear it open. On this principle was derived the great success which attended the operations of that celebrated lithotomist, Klein.

That it is the forceps which is the great agent of destruction, in the larger number of cases, is clear, from looking carefully over the sizes of the stones extracted by the late Mr. Martineau. He encountered no very desperate cases,—he was thus as fortunate as he proved skilful.

Out of eighty-four cases, the two largest stones weighed each four ounces only, and one of these patients was lost. Why? Because the forceps had too much to do in the extraction. When the stones were small, which in his great experience was remarkably the case, the forceps had very little to do. Hence his extraordinary success, and the detection of the true source of destruction.

Mr. Martineau, therefore, could never be justly quoted as an authority for violence in lithotomy. He seldom had occasion, from the size of the stones, to employ it; but when he had, he lost his patients like other surgeons.

There are, however, other sources of injury besides the forceps. Such as I have seen, from that and other causes, with all the failures which have occurred within my observation, shall now be faithfully narrated."

### CASE 1. *Fatal Abscess of the Pelvis from a lacerated Bladder by the Forceps.*

"A Sexagenarian from the country, tall, and very little worn in constitution by the presence of a large stone, which he had carried some years in his bladder, came under my care to have it removed. It was my maiden operation, and I was surrounded by experienced friends. From repeated examinations made in the rectum, and by sounding, it was evident that the stone was beyond a common size, and preparations were made accordingly; the muscles were fairly and freely cut, and the prostate gland divided by a full sized gorget. The stone-breaker was at hand. It was not difficult to lay hold of such a stone; the difficulty was in bringing it through its narrow channel with safety to the patient. I made gentle efforts in the proper direction, put my finger upon it between the blades of the forceps, in the rectum, and this examination assured me that it would never pass, without more force and laceration than was consistent with the patient's safety, and with my notions of the mode in which this operation should be performed. In vain was the opening into the bladder enlarged by the bistoury, and a more powerful exertion made;—the stone would not pass.

I looked round for the stone-breaker, I begged that it might be handed to me. 'My dear Sir,' with a pinch on the elbow, 'try again,' was the reply on one side. I did so, reluctantly; another more powerful, though unsuccessful pull was the consequence, and again I entreated imploringly for the stone-breaker; 'Nonsense, don't be afraid, I have used ten times more force than you now do,' was the answer from another side, (it was true, but his patients rarely survived;) one effort more, indeed, succeeded in bringing forth the stone, which was of the mulberry kind, and weighing about five ounces and a quarter, and after the patient had been upon the table three quarters of an hour. The last adviser was not a little proud of so speedy a proof of the soundness of his advice,—but he should have waited the result.

The shock of the operation the hardy veteran sustained; its immediate danger passed away,—but he soon fell off, and ultimately sunk under irritative fever, at the end of the fifth week from the date of the operation.

The irritation was a large abscess in the cellular membrane, between the bladder and rectum, and which doubtless arose from the injury done to the prostate gland and neck of the bladder, which were in rags, or fringes, bedewed with pus. The result of the foregoing case was of service, though not to the patient. I became particularly cautious of committing the slightest violence beyond what was absolutely necessary, rather cutting even the bladder, than allowing of any force in extracting the stone from it; and the effect was, that the next nineteen operations were successful ones. The stone, in this case, was too large to be removed with certain safety, though there would have been more chance of success with less violence. We may call for, as was done in the foregoing case, and then look at stone-breakers,—but to use them is, perhaps, quite a different matter. Mr. Earle's is the best. The lithontrite appears to be inadmissible, from its want of power over large stones." 83.

CASE II. *Fatal Peritonitis from a Lacerated Bladder.*

In the following case Mr. Fletcher conceives that violence was the cause of death. The preceding case was the first of his which proved fatal, and this was his second and ultimate loss. The patient was a young man, twenty-two years of age, and a good subject, for no tendency to organic disease could be detected in him. The following note may looked on as explanatory.

"No man should cut for the stone when he is ill; the feeling of lassitude, weakness, and want of decision, will creep into the operation. A slight oversight in the design, or defect of vigour in the execution, are quite enough to give a fatal turn to its termination. To-day I was not sufficiently alert, the gorget was overlooked, it did not cut well close to the beak, nor was it broad enough for a large adult, so that the right side of the prostate was not completely divided. The muscles too, in the deep hollow between the ischium and anus, were not sufficiently or decidedly cut, so that a straitened channel was left for the exit of the stone. Both these original errors were amended, though feebly and efficiently,—illness was at the bottom of it,—the division of the right side of the prostate was completed, and the bridles of muscles touched with the knife. These subsequent corrections were not enough to prevent more violence being done than should be permitted in this operation. It was ten minutes before the stone was extracted; and though I have seen infinitely more rough exertion employed, without harm in the result, yet do I fear for this poor fellow." 84.

The stone weighed four ounces. In spite of early attention and active treatment the patient died of peritoneal inflammation five days after the operation. The angle of the left division of the right side of the prostate gland was torn, shewing that its division by the prostate had not been quite accomplished. There was some pus in the cellular membrane in its neighbourhood, and the small intestines were glued together by active peritoneal inflammation.

CASE 3. *Immediate Death from Extraordinary Violence in using the Forceps.*

A healthy, middle-aged man submitted to all the preliminaries of the operation with an air of great determination. The operator made his incisions well, reached the bladder, felt the stone, and quickly introduced a large pair of forceps. Difficulty was experienced in getting them in, a large stone was grasped, and now still more difficulty was encountered in getting them out. Violent efforts were made, great violence was used, and that not apparently in the best direction, the tugging was maintained for nearly two hours, and then a stone weighing more than five ounces was extracted. During the earlier part of the operation the patient continued to utter shrieks of agony, but at length they sank into a moan, and when the stone was shewn him it was doubtful if he saw it. He expired in a few minutes after being carried to his bed. The body was not examined.

"Upon looking at the gorget, I thought it certain, that it could not, from its small size, have completely divided the left side of the prostate, moreover, it cut only on one side, so that room was lost on the right side of this gland. The operator too, having seized the stone, appeared to be unwilling to part with it, fearing it would be difficult to find again; although he must have felt a stricture or binding upon it, which would require great force to overcome. He appeared to be mentally whispering to himself, 'if I let it go I may not get hold of it again, and it shall come now it is in my power,' and with this wrong understanding of the principles of this operation, the fatal pulling was continued." 86.

If there is any considerable sense of stricture at the prostatic opening when the stone is to be extracted, the latter should be relinquished, the extent of the incision ascertained, and the latter enlarged according to circumstances. After all the presence of a large stone is unfortunate.

Case 4. *Fatal result, from Injuries to the Bladder in attempting to Extract the Remnant of a Stone.*

A young man, eighteen years of age, had for some years been labouring under the symptoms of stone in the bladder, and placed himself under a good operator for its removal. The operation was done well, but unluckily the stone broke in the gripe of the forceps, and after a long trial of means for its extraction the fragment of stone was left in the bladder. For a few days there were merely some slight pains about the loins, and a little tenderness about the lower part of the abdomen. But soon irritative fever shewed itself, and the patient sank a fortnight after the operation. The difficulty of seizing the stone probably arose from a portion of the bladder having contracted upon it, for after death the stone was found loose at the bottom of the bladder. The operation lasted nearly two hours. On dissection, the right kidney was found in a state of suppuration; the left contained fetid serum. The ureters were enlarged, their coats thickened, and highly vascular. The coats of the bladder were full half an inch in thickness; its internal surface every where covered with black fetid mucus, except at the fundus, which appeared more healthy but inflamed. A piece of stone was found in the lower and back part of the bladder. The prostate gland was hardly any thing else than a mass of fetid matter.

Mr. Fletcher thinks it unquestionable that the young man died from the effects of the long-continued violence of the operation. In similar cases, where so much difficulty is experienced in extracting portions of calculi Mr. F. thinks it better to leave them in the bladder, and take the chance of a natural exit. Moral courage is the thing which is required. The spasm which holds the stone is more likely to yield, when the coarse attempts at its extraction are desisted from.

CASE 5. *Death from Sounding for a Stone.*

A boy, six years of age, was twice sounded for stone by the surgeon of a hospital, and, the stone having been felt, the day was fixed for the operation. The child was tied and sounded by the operating surgeon, but he felt no stone. The consulting surgeon took the sound and used it rather roughly with the same ill success, and another and another visitor did the same. The surgeon now interposed and the boy was carried to bed. He complained that his belly ached, active peritoneal inflammation followed, and on the fourth day from the sounding the child died. On examination the inner lining of the bladder was found highly inflamed, and its peritoneal covering, at the fundus, was glued to the intestines, which were, on all sides, inflamed and smeared with lymph.

CASE 6. *Death from continued violence in seeking for a small Stone, after the Bladder was opened.*

"A surgeon made his way very skilfully into the bladder of a little boy, in which a stone was distinctly felt, and he could, on the introduction of his finger, occasionally touch it. The forceps were introduced, with closed blades, and the point of the instrument every now and then would strike upon the stone, but when the blades were opened, and the surgeon endeavoured to grasp the stone, he found it constantly eluding their gripe, or slipping out of them. The operation continued in this way for nearly half an hour, the patient complaining greatly of how much he was hurt; but at length the forceps seized the stone securely, which was extracted with the utmost ease,—for its size was singularly small.

The boy was put to bed, struck heavily by the operation,—he was cold, and somewhat torpid, with a very feeble pulse;—and from this state he never recovered, although cordials and opium were given to him.

There was some, but very slight, tenderness of the abdomen on the following day; the patient was bled, and took opening medicine, and, of course, treated for peritonitis,—but he died on the fourth day from the date of the operation.

On inspecting the body, no signs of inflammation could be detected in the bladder or peritoneum,—all was pale and healthy. The bladder was, indeed, thickened, but this must have been the work of times past.

That this boy perished from the effect of a long-continued and worrying operation upon his nervous system is sufficiently clear from the dissection, and also from the circumstance that he never rallied, but remained cold, with the peculiar torpid and fatal heaviness upon him, which is seen when little children are sent into hospitals with dreadful burns about the trunk of the body. The destruction of the power of the brain and nervous system, by the violence of the shock, is the cause of death in both instances.

There is much variety of opinion as to the propriety of bleeding after lithotomy. Some practise it, and strongly recommend that it should be had recourse to upon the detection of any tenderness about the abdomen, notwithstanding a feeble pulse. There was certainly tenderness in this case.

For my own part, and from experience it is stated, I should be slow of bleeding after lithotomy, whilst evidence of decided prostration of the nervous system remained, in the shape of languor, indifference to external objects, sleepiness, and diminished temperature, even should some tenderness be present.

Above all things, it is presumed, the surgeon should be cautious in bleeding children under these circumstances, and especially gentle in his treatment of them during operation. It is very true, that in lithotomy children do better than grown persons; more recover. But this is to be accounted for on the ground that the operation in them is comparatively nothing. The stone is always small, and the operation throughout is much easier and quicker to perform, and, therefore, the little patient has less to endure. For the explanation of the greater success of lithotomy in children cannot be in their superior power of bearing suffering. Their irritability is greater, their nervous system sooner excited: and hence, in dentition, and in irritations within the alimentary canal, are we often obliged to witness the most distressing sufferings. It is probable that the majority of deaths from lithotomy, in young children, is from the blow inflicted on the nervous power by the necessary severity of the operation, and not from inflammatory actions set up by it." 93.

Case 7. *Fatal Peritonitis from Laceration of the Bladder in the Operation.*

These cases are related in such a manner that it is difficult to condense some of them, and preserve at the same time that record of symptoms which constitutes their value. We must give the following in the original words. The case occurred to Mr. Fletcher's predecessor, well known as an excellent and successful lithotomist.

"I operated, in lithotomy, on a tall, well made man, twenty-nine years of age, who had been upwards of two years suffering severely from the stone, but, though somewhat emaciated, and apparently of an irritable constitution, he appeared by no means an improper subject for the operation.

In the introduction of the conductor I met with some resistance. I am not certain whether it was from my not having divided the muscles with sufficient freedom, and consequently the urethra close to the prostate gland; or from my pressing the beak of the conductor with too much force against the groove of the convexity of the staff. However, I resumed my knife, and divided the urethra and a very small portion of the prostate, and then the conductor passed with ease. The stone was readily laid hold of by the forceps, *but the resistance was so great, that it was not without spending some time, and using much violence, that I could extract it.*

The man bled very profusely, immediately after the coming away of the stone. However, after his legs were united, and his thighs brought together, the hemorrhage very much diminished, and he was sent to bed. He complained immediately of great pain just above the pelvis. After he was put to bed an opiate was given to him. A very little weeping of blood from the wounds was observed. An hour after I left him I sent my pupil to examine if it continued. He brought me word, that there was a continuance of the hemorrhage in an increased degree, and that the man was in great pain. I immediately went to him, and found him in the condition described. I perceived that the discharge, though thin, was not urinous, and, therefore, I concluded that it was chiefly the serum oozing from coagulating blood, and consequently that there was a considerable lodgment in the bladder. I gently dilated the wound, and had the mortification to find my opinion confirmed. I brought away a large quantity of coagulated blood. The poor man expressed a sense of great ease, but then the blood flowed most copiously through the external wound. I introduced my finger covered with lint, and took other alike ineffectual means to restrain the hemorrhage. Dr. Cheston was so obliging as to lend me his assistance. I tried a canula covered with lint dipped in astringent liquids, and a variety of methods, with little or no benefit. The man lost an immense quantity of blood. At length we left the wound to itself, and applying a solution of sal ammonia cold over the belly, and over the wound, and rags wetted in the same to the hypogastrium and between the thighs, the hemorrhage was entirely suppressed. The pain, however, continued. It increased during the night. The next morning his belly was sore, and somewhat tense. He was immoderately thirsty. He was sick, and troubled with frequent and feeble eructations.

He continued to grow worse and worse, and died on the fourth day.

On dissection there was a *considerable laceration of the bladder*, in a variety of directions, though none of them extensive.

It seemed that the hemorrhage was from the bladder, but this we could not fully ascertain. The peritoneum was generally inflamed, but there was no other unnatural appearance.

I do not, upon a review of this case, see any thing which could have been done in addition to, or variation from the means used, unless it was that I should have made a more free wound in the muscles, *if I had been aware of the size of the stone.* For though the resistance was apparently altogether in this bladder, yet it is probable that the wound in that organ would have been sufficient, and the opening would have dilated, if I had, by a very free and large incision through the muscles, removed all support to resistance, which the bladder itself had given." 95.

Mr. Fletcher concludes that, though the hæmorrhage was great, it had no share in producing the death of the patient. Of this we are not so sure. Granting that he died of peritonitis, still peritoneal inflammation supervening on such loss of blood, must be pretty inevitably fatal. But, no doubt, the constant flow of blood into the bladder, and the continuance of irritative means employed for its suppression, must have assisted in the production of peritoneal or cellular inflammation.

Case 8. *Peritonitis from Violence in the Operation—Ultimately fatal Abscess of the Pelvis and Kidney.*

This operation was performed by the same lithotomist, and, unwilling as we are to indulge in quotations, we really cannot abbreviate it.

"I cut a boy of ten years old. He lost a good deal of blood in the operation, from a branch of the pudica. In the evening his pulse was frequent, but in other respects he was doing well. In the night he complained a little of his belly. The next morning, his pulse being frequent, and his belly somewhat tense, I took from him about eight ounces of blood;—by the time of his having lost it, his lips became pale, and he shewed signs of faintness, and soon after was a little sick, and a profuse sweat broke out. His pulse grew a great deal quicker, and smaller, and it is scarce credible how rapidly the peritoneal affection increased. Before the bleeding, he could bear his belly to be struck gently, or to be handled without pain. It was not much swollen; but within half an hour after the blood had been drawn, the abdominal region became universally very much swollen, tympanitic, and exquisitely sore to the touch. He appeared to be in a great deal of pain, and his countenance grew expressive of great distress. His pulse was uncommonly quick and feeble, probably at least 180. He had had no stool since the operation. His urine was freely discharged through the wound, and some had passed through the urethra.

Clysters were given him, a blister was applied to the false ribs on one side, and a sinapism to the other. Warm fomentations of poppy heads, decoctions, and crude Sal Ammoniac. were used to his abdomen for an hour or two; but they seemed to do mischief, and to increase the pain and the swelling. Infus. Senn. cu. P. I. was given him, and afterwards some Ol. Ricin. after using the fomentations for two or three hours. Finding they afforded no relief, I changed them for Spt. Vin. Camph. cu. Tinct. Opii, applied cold, which seemed to lessen both the soreness and the pain. The next day he was better, the soreness of the abdomen was lessened.

The twenty-first day after the operation he died. In the course of seven or eight days he was very much amended, having natural stools, and a great part of the urine passing through the urethra. His abdomen being free from pain, but his pulse was always quick, his appetite did not return, he had generally upon him a thirst, and his tongue was in general whitish; nor had his abdomen a natural feel; so that it was pretty evident there was some latent mischief, though of what kind I could not tell; beside, he every day grew more and more emaciated. His nights, though not painful, were disturbed. The nurse observed that his water was often whitish.

Upon opening his body, I found the bladder contracted to a very small size, so that its cavity would not have contained more than two or three spoonfuls of urine. It was nearly half an inch in thickness;—red spots, as of inflammation, appeared here and there on its mucous coat, which, however, seemed in no place to be ulcerated.

The wound which was made by the operation, and which, probably, had been somewhat increased in magnitude by ulceration, *(for the sides of the wound had sloughed,)* appeared to be larger than I expected, or intended it should have been; for the prostate gland was completely cut through, and the incision was continued quite through the neck of the bladder, the orifice of one of the seminal ducts was obliquely wounded, notwithstanding the great care which I took in directing my prostate knife.

One kidney was very little altered from a natural state, its pelvis, however, and the beginning of the ureter, were very much enlarged. The other kidney was merely a leathery cyst, full of matter, but a great deal diminished from its natural size. In the neighbourhood of the bladder, the intestines were adherent one to the other, and appeared to be considerably inflamed. Upon tearing the adhesion through, the posterior part of the pelvis was found full of thin pus, probably there was a pint and a half of that fluid. The sacrum was even bared by its action, and the rectum was loosened from its attachment to it.

The foregoing eight fatal cases, with one other, form the whole number of deaths that have been witnessed by Mr. Fletcher, out of fifty-nine operations that have been performed within his knowledge. He believes that in all, with the exception of the first and perhaps the third case, the loss of life arose entirely from what may be deemed an unnecessary violence. If the stone be very large, and the lateral operation made choice of, it is impossible to avoid that violence which Mr. Fletcher so earnestly deprecates. Under these circumstances he would adopt Professor Vacca's first or last recto-vesical operation, an operation which he justly considers as not to be placed in competition with the lateral operation for calculi of moderate dimensions.

In the four following cases the result was not fatal, although it was nearly so.

CASE 9. *Peritonitis from long-continued Attempts to find the Stone—two Stones—one sacculated—a portion left behind.*

Mr. F. performed lithotomy Feb. 19th, 1817, on a man seventy-three years of age, of a good constitution. There was much difficulty in feeling the stone with the staff, and many indiscreet attempts were made to do so. There was nothing remarkable in the operation, excepting that, besides a large stone, the bladder contained a smaller one encysted immediately above the pubes. A long pair of dressing forceps broke off the point, and the remainder was left behind, which the nail of the fore-finger could feel on a level with the sides of the cyst and the bladder. No further attempts were made to dislodge this stone. In the night there was severe pain, with some swelling just above the pubes; the skin was rather hot, tongue furred, pulse hard and about 100. Thirty leeches were ordered, and when these had ceased to bleed, cold rags were to be constantly applied. In the following evening the pain was much diminished, the swelling gone, but there were no stools and some magnes. sulph. was prescribed. On the third morning it was reported that he had vomited twice in the night; the belly was more swollen, and very tender; the tongue furred; pulse 111. *V. S. ad* ℥xviij.—*hirud.* xij. *abdomini*—*enema purgans.* When the leeches had ceased to bleed he was placed in the warm-bath, and afterwards a large blister was applied. In the evening the symptoms were relieved, the pulse was 45. A glyster, with some infusion of senna, and sixty drops of laudanum was ordered, and on the following morning the improvement was even greater. On the fifth day, in the evening, the pulse was 88, sharp and strong, the tongue was dry, he was more restless. *V. S. ad* ℥xvij.—*hirud.* xij. *epigastrio*—*mag. sulph. omni horâ.* From this time he recovered gradually and was ultimately discharged cured. When last this patient was heard of he was well, and suffered no inconvenience from the sacculated stone in the bladder.

Case 10. *Inflammation of the Bladder from Sounding—Hæmorrhage during the Operation—Bladder full of Stones.*

Charles Pride, æt. 50, was admitted into the Infirmary under Mr. Fletcher, with stone in the bladder. He was twice sounded, and each time, after the operation, he complained of great pain and tenderness in the lower part of the belly, vomited repeatedly, and his urine deposited a great quantity of mucus; these symptoms were relieved by free leeching—the warm-bath—purging. At the patient's urgent request Mr. F. performed the operation. During the operation the transversa perinæi bled nearly a quart. On cutting into the bladder Mr. F. found two large stones in it, and it was necessary to enlarge the wound in the bladder to an unusual extent. Both stones broke under the forceps, and were extracted in less than half an hour; they weighed about six ounces. After the operation the man became low and cold, and required some brandy. Next morning he began to vomit bilious matter, had constipation, pulse 120, feeble, tongue furred and white. He rejected effervescing draughts, &c. In the evening he complained of slight pain and tenderness at the bottom of the belly, he vomited during the night, and twenty leeches were applied next morning. In the afternoon his pulse was imperceptible, he was ordered brandy repeatedly, and in the night he had stools. He recovered.

This patient subsequently returned with fresh symptoms of stone in the bladder. The operation was performed, the stone found to be enclosed by a contraction of the bladder above the pubes, and it could not be dislodged by the forceps. With the tip of the fore-finger of the left hand as a guide the handle of the scoop was conducted to the stone, by gently depressing the handle the extreme point was inserted over the calculus, and with a few gentle efforts the stone was turned out of its sacculated position, and fell to the bottom of the bladder, whence it was easily removed by the forceps.

CASE 12. *Hæmorrhage during and after the Operation.*

While Mr. Fletcher was operating on a healthy young man the stone broke under the first gripe of the forceps. The fragments were not extracted under half an hour, during which period hæmorrhage was going on. The vessel lay deep under the pubes and bled rather profusely. A large rectum bougie covered with oiled lint, held by an assistant, suppressed the bleeding, but in seven hours after the operation the instrument was displaced, the bleeding recurred, and half a pint of blood was lost. The instrument was replaced. On the third day it was withdrawn, and on the sixth hæmorrhage took place to the extent of three pints. The instrument was carefully introduced, but removed, from its producing extreme soreness, on the following day. No further hæmorrhage followed, and the patient was dismissed cured.

CASE 13. *Irritable Bladder— Violence in operating—Abscesses in all possible directions.*

Charles Matthews, æt. 12, was admitted May 23d, 1822, with symptoms of stone, and mucous sediment in the urine. On the 25th he passed a small stone. On the 4th June he was sounded, and a stone distinctly felt. In the evening he had great pain, and some tenderness in the belly, and next day there was more mucus streaked with blood, and great frequency of micturition. On the 3d July he was again sounded, and the same symptoms followed. On the 15th the operation was performed. The transversalis perinæi required to be tied. The opening in the bladder was made by the gorget, but not being large enough, a little violence was exercised by the forceps, and the stone extracted. It was large, soft, similar to sand-stone, with volatile effluvia. *Tr. opii*, ♏xxx. He went on pretty well till the 17th, when there was pyrexia, with slight tenderness in the lower part of the abdomen. Stools were procured by castor oil, but the symptoms continued next day, and were relieved by a warm bath. On the

2d August he had rigors, and the wound now began to discharge profusely, matter flowing from the wound, and apparently through the anus, on pressure of the belly. After this several collections of matter took place, and burst externally in several places in the region of the bladder above the pubes. His health for a time was indifferent, but by July 1823, when he was discharged, it was re-established. At this time the discharge by the original wound was very small, but the urine still passed occasionally through those above the pubes.

With the preceding the cases of lithotomy are concluded. It will have been perceived that the point to which all our author's observations and precepts tend, is the avoidance of violence in the operation, the advantages of cutting over laceration of the parts in the extraction of the stone. We have said, and we scarcely need repeat it, that on this point experienced surgeons are not yet agreed. Mr. Brodie protests against complete section of the prostate gland; he would rather tear to a certain degree than cut, in order to shun the dangers of cellular inflammation. We trust we shall be pardoned for conceiving that this point requires further investigation, and that investigation can scarcely fall into better hands than those which have already been engaged with it. To pass to another subject, we think that the following remarks on lithontrity are not uncalled for at the present period.

"With regard to the modern practice of destroying the calculus, of boring, splitting, and hammering it to pieces in its soft and tender residence,—time only can discover the real value of the measure. On a first view,—it must be admitted that first views are frequently erroneous,—and on reading some of the foregoing cases, the reader may probably conclude, that if it were possible to select another spot, he would not choose the human bladder as a desirable situation for the operation of stone-breaking. They show that the mere irritation of a sound,—and of the closed forceps, in searching for a stone in the bladder, is sufficient to excite fatal inflammation in that organ,—and, in cases where no evidence existed of previous disease in the bladder or prostate gland, which would have pre-disposed those parts to fatal disorganization, under ordinary operating circumstances. Of course the work of death was effected by the irritation of the instruments employed in lithotomy. Will the lithontritic instruments, under similar circumstances, prove less irritating? Three cases out of seven, in one gentleman's practice, perished from inflammation of the bladder excited by them. Picked cases, paraded even before our best surgeons, do not furnish, either in kind or degree, the evidence necessary to decide upon the merits of lithontrity, and how far this innovation is likely to supplant or supersede the ancient and successful practice of lithotomy. Great and unbiassed experience of the most unquestionable kind,—faithful and honest narration of facts, pleasant or unpleasant,—of successes and *failures*, fairly intermingled alike,—with a love of truth,—can only decide the question. Evidence too, selected or not selected, should be taken with great caution, when proceeding from interested sources. Lithontrity is chiefly in the hands of those who either get their bread by it, or wish to do so; and it is too much to expect that Nature should be the first to cry out against the gratification of her first wants. There is enough of evidence, however, to shew that the new operation bids fair to become a valuable auxiliary, if not a principal, in the treatment of calculi in the bladder; but the proper persons to mark the boundary of its merits, are those who get their bread from other sources. Surgeons generally, and especially hospital surgeons, whose operating habits, and greater experience in calculous affections, especially fit them for the important office of umpires, should no longer hesitate; for it is unquestionably both a matter of surprise and regret, that, up to this moment, not one case of lithontrity has been performed by a British surgeon, in a British hospital." 111.

### II. ON SPASM OF THE GLOTTIS.

Mr. Fletcher observes that it may appear strange to write on the origin of an affection which some may believe not to exist. But as life may sometimes be suddenly extinguished by a sore throat, an abscess, or a wen, it becomes of consequence to determine the exact mode by which that process is effected, and to discover the several causes or particular affections which produce it. Some eminent men have believed that suffocation, from affections of the throat, is often occasioned by spasm of the glottis; and arguments and facts have been adduced in favour of the notion. The stricture, like that of the urethra, œsophagus, and rectum, is probably a spasmodic stricture, a sympathetic affection. Its cause is to be looked for in diseases of the throat which must form the objects of our study. The spasm of the glottis may be severe, or it may be slight, in the first case proving rapidly fatal, in the second occasioning slight convulsive catches of the breath. The complaint is not accompanied by fever, unless this be produced by any of the inflammatory affections which excite the spasm itself. The following symptoms were noted down by our author soon after leaving the bedside of a patient.

"When first seized with spasm of the glottis, the patient starts up suddenly, tossing his arms in wild affright; an expression of terror, as if attacked by some dreadful enemy within, sits upon his countenance; the eyebrows are raised over balls that are starting from their sockets; the shoulders rise and fall, as with an open mouth, and incredible exertions, air is drawn through the nearly obstructed tube, with a singular and alarming sound. Not a word is uttered, enough of the understanding is left in this moment of terror, to induce a belief in the unfortunate patient, that one would be fatal. But as the violence of the spasm subsides, he tells you in monosyllables as well as he can, that he has been nearly choked. This communication is sometimes made with a voice that causes you to start;—a deep, unnatural growl rises from the throat; or it is a fearful broken whisper, that still bespeaks terror, though now on the decline. As this terror continues to fade, the expression of the whole man assumes a different character. Apprehensive of a return, he seizes the bed-clothes with his hands, which before were tossing in the air, that he may not be taken unprepared, and with one or the other, he will occasionally point to the thyroid cartilage, as the seat of all his sufferings. A pale, haggard, and subdued countenance, on which are seen a few drops of cold perspiration, is before you; the mouth half open; the breathing yet hard. The eye-balls indeed have in a measure retired within their sockets, but the eyes themselves, as the understanding rallies, wear a mingled expression of keen, watchful intelligence, which follows your every movement, with restless anxiety. On your countenance and actions, are bent all the powers of the sufferer; a steady gaze meets you every where; if the bearing be calm and determined, there is hope, but if you betray a wavering countenance, and an inclination to reach the door, you will be detected, and your patient, should he recover, will put you down as nobody, or as one of little value in the hour of danger and difficulty." 3.

Case 1. *Fatal Spasm from an Ulcer in the Œsophagus.*

Abigail Tarret, æt. 18, was admitted into the Gloucester Infirmary, having complained for some weeks previously of a pricking pain and difficulty in swallowing, and difficulty of breathing, especially at times. There were constant pain in the throat, neck, and side of the head; some swelling, with hardness and rigidity of the muscles of the neck, and lymphatic glands; great pain from the least movement of the head; incapability of swallowing solids, and much difficulty in swallowing liquids; large quantities of mucus in the throat, which were expectorated with difficulty. In about a month after admission she died suddenly, the breathing having previously become alarmingly impeded. The only morbid appearance found on dissection was a large ulcer in the œsophagus, about an inch below the situation of the cricoid cartilage.

Case 2. *Fatal Spasm of the Glottis, from Bronchocele.*

—— Takell, æt. 14, was admitted into the Gloucester Infirmary for a bronchocele which had existed for seven years. Latterly her breathing had been occasionally much embarrassed, and the tumour had been tender and painful. On examination of the tumour she complained of much pain, and inspired with difficulty. Some leeches were applied, and on every occasion the same train of symptoms followed; she sprang up in bed, drew in the air with a laborious exertion, and her countenance assumed a painfully haggard expression. A seton was introduced. The remainder of her life was passed in a succession of struggles to get air, and, as swallowing exasperated them, she attempted to take food no more, and died before the preparations for bronchotomy could be completed.

On examination there was found a cyst, which arose from the centre of the right lateral lobe of the thyroid gland, and contained about a small teacupful of a fluid resembling ink. The lateral lobes themselves were much enlarged, the right most so. The larynx was perfectly healthy.

Case 3. *Fatal Abscess of the Glottis, from a Chronic Abscess of the Pharynx.*

A middle-aged man had laboured for some time under great difficulty of breathing and swallowing, with soreness and constant pain in the throat. His respiration was deep and hoarse, not unlike the barking of a dog. There was nothing unusual in the fauces, save that the tonsils were remarkably far back and separated from each other. One morning his breathing suddenly became extremely difficult, and in half an hour he died like one suffocated. On examination, there was discovered a chronic abscess deep in the pharynx, situated about two inches from the fauces, and containing about two ounces of pus. This very much lessened the size of the canal through which the food was to pass, and also made great pressure on the larynx, though it did not actually close it.

Case 4. *Spasm of the Glottis from Cynanche Tonsillaris, with Abscess.*

In May, 1829, our author was summoned to see Mr. S. of Ashleworth. He found him exhausted, lying semi-recumbent, breathing noisily through the nose. The fauces were unusually swollen, the velum pendulum palati and tonsils advanced into the mouth, forming a complete barrier, and closing entirely the communication between the latter and the pharynx. He had had a sore throat for four days, had been bled and blistered, and for the last two days could neither speak nor swallow. Shortly before Mr. F. was sent for, the patient had been suddenly attacked with violent convulsive difficulty of breathing, which had threatened strangulation, and only ceased immediately before his arrival. Into the prominent part of the velum our author plunged a pharyngotomos, and a torrent of pus burst forth; the tonsils and velum were scarified freely. Mr. S. slowly recovered.

The next case is that of a girl affected with chronic enlargement of the tonsils to a very considerable degree. On the supervention of any more acute inflammation from cold she would suddenly be attacked with paroxysms of extraordinary difficulty of breathing, the eyes starting from the head, and her respiration being accompanied by a peculiar sound. These paroxysms would only occur when she slept. She was sixteen years old, and had never menstruated. Leeches, &c. relieved her.

Case 6. *Suffocation from a Venereal Ulcer of the Throat.*

——— Joy applied at the hospital for a sore throat. His voice was a growl, he had great difficulty in swallowing, and sometimes of breathing, especially when he lay in bed. The breath was very offensive, but no disease in the throat was visible. He frequently expectorated thick matter, occasionally mixed with blood. There was an ulcer on the head. He had recently had sores on the penis and buboes, which had healed under mercury. He was recommended to enter the hospital, and exerted himself a good deal during the day to procure an admission letter. In the evening he was admitted, but during the night he was suddenly seized with spasmodic difficulty of breathing, and died immediately after swallowing, with great difficulty, an opiate. A large sloughy ulcer occupied the lower portion of the back of the pharynx, and had extended itself to the back of the larynx. The rima glottidis was free from disease, and its channel of natural size.

Case 7.—*Spasm of the Glottis from Phlegmonous Inflammation of the Neck.*

"I was requested to drive with speed to a gentleman, who was said to be suffocating. I had scarcely entered the house, when it was reported, that the violence of the attack had somewhat subsided, though the patient was still gasping for breath.

He was sitting nearly upright on the bed, and on my name being announced, he fixed his gaze anxiously upon me, as if to inquire what relief he might expect, from a close scrutiny of my bearing and personal appearance. The expression of the countenance had also the wild horror of one who had just escaped some frightful personal danger, and who was yet momentarily expecting again to be overtaken by it.

The Spasm of the Glottis was here plainly to be seen, in the occasional convulsive catches of his breath, though its violence had in a measure passed away.

He grasped, as he sat, the bed-clothes with both hands, and his mouth was open, drawing in air with great difficulty, his shoulders rising and falling as he did so, the eye-brows raised, the balls starting forwards, and a cold dew upon his pale face. As I approached the side of the bed, he let go the clothes with his right hand, and pointing to the front of the larynx, said in a hoarse and husky whisper, 'I shall be choked.'

This gentleman had been subject to sore throats, indeed he had lately had one, the effect of cold on a night exposure.

At present there was no vestige of any inflammation about the interior of the throat, though he now swallowed with difficulty: but on the exterior there was a general swelling across its middle, more especially on the left side, a little below the inferior cornu of the thyroid cartilage, deep and immediately over the carotid. The swelling had ascended high in the neck, making it look generally of a colossal size. At the point near the thyroid cartilage there was a redness, tenderness to the touch, and pain, marking the presence of phlegmonous inflammation.

The pulse was quick; he was hot and thirsty. Fifty leeches were applied to the inflamed parts, and a strong purgative given.

Three hours afterwards I visited this patient. The bleeding had been profuse, and his respiration was infinitely more calm; though once, and for a minute, since my last visit, he struggled so much for air, that his attendants thought he would have gone. This character, indeed, had prevailed throughout; respiration always difficult, but at times the convulsive struggle and stricture about the larynx, frightful.

I watched the mode of breathing for nearly half an hour; and being satisfied that the regular difficulty, mingled now and then with spasm was greatly diminished, and that I saw no violent spasm of the glottis, I left, with instructions to be sent for, should the respiration become worse.

I heard no more of the progress of this patient, till I visited him eight hours afterwards. The breathing was nearly natural, the swelling in front of the throat greatly reduced, and the skin become pale. There appeared, however, a little hollowness of the point, which was red, near the inferior cornu of the thyroid cartilage.

The spasm of the glottis did not return: but a day or two afterwards, I understood that the patient brought up some offensive matter, making it probable that suppuration in the cellular membrane of the neck had taken place, and found its way into the pharynx." 14.

### Case 8. *Spasm of the Glottis from Laryngitis.*

"A medical friend of mine, about fifty years of age, sent for me in the night, with a message that he feared he should be suffocated. I found him sitting up in bed, an expression of alarm on his countenance, and breathing with great difficulty, though regularly, that is, without those sudden exacerbations which indicate spasm of the muscles of the larynx. He however said, that before my arrival he had a more than common difficulty of breathing, that terrified him exceedingly, and so fearful was he of its return, that he would not permit his daughter to leave his bedside, where she was stationed to prevent his going to sleep, the fit, as he called it, having taken place during that period. His pulse was quick, and full; there was thirst, with some cough, and he brought up, occasionally, a quantity of mucus. He had been a good deal exposed of late to the night air, in his practice of an accoucheur, had been ill about two days with soreness of the throat in swallowing, and some difficulty of breathing, and fever, but nothing in degree to what he now suffered. He referred all his distress to the larynx, where he placed his finger; he complained of pain there, but especially of a sensation like the drawing of a purse-string, to close the channel of the purse: 'but,' said he, characteristically, in a croaking, broken voice, 'I suppose the string is not yet drawn very tight, when it is I shall be no more.'

As the fauces indicated no inflammation, and but very little redness, and that only visible when the tongue was well kept down on the lower part of the back of the pharynx, and as the boundaries of the neck were free of all irritations which might affect the glottis sympathetically, and as the patient had taken cold, had cough, and symptoms of fever, together with a regular difficulty of breathing, independent of all spasms, and also expectoration of mucus, I ventured to believe, (more especially as he complained of regular pain under the thyroid cartilage,) that this was a case of inflammation of the mucous lining of the larynx, and perhaps of the lower portions of the pharynx, which excited the spasmodic action of the muscles.

He was bled largely from the arm, and forty leeches were applied as nearly as possible about the thyroid cartilage, to the spot where he complained of pain.

Under these active means the symptoms gradually faded. He retained his daughter however for a long time to prevent his sleeping, a painful and self-denying resolution for an exhausted man, only to be accounted for, from the deep impression made on his memory by the suffocating nature of the spasm, which seized him during sleep.

To this day the impression of terror is as fresh as ever in this gentleman's memory; the fear of taking cold, and of a return of the attack is constant, and in consequence I believe he retired from his business as an eminent accoucheur." 16.

Case 9. *Spasm of the Glottis from an Elongated Uvula.*

A steward of Colonel Berkeley's applied to Mr. Fletcher on account of occasional attacks of spasm of the glottis, which for some years he had been accustomed to experience during the night. The attack occured during sleep, and impelled him to rise suddenly to seek relief, which always followed his change of position. On examination of the fauces, there was found no enlargement of the tonsils, but the uvula was remarkably elongated, its point lying horizontally on the tongue; the whole of the fauces had a chronic deep red, and relaxed appearance. The gentleman had an almost constant feeling of something sticking in his throat, and suffered from the ordinary symptoms of indigestion. He would not consent to removal of part of the uvula, and was put upon tonic treatment, with powerful local astringents.

The foregoing are not all the cases related by Mr. Fletcher, but they are the most pertinent and impressive. We pass therefore to his observations on the disease, and on its treatment.

"It will be seen, in the foregoing cases, that the stricture of the glottis, the effects of irregular actions of its muscles, and which terminated in many of them in instant death, was produced by this interesting and important canal, sympathizing with the following distinct affections, more or less distant from itself.

1. From the irritation of acute inflammation of the mucous membrane of the fauces, where it covers the tonsils, velum pendulum palati, and tonsils (cynanche tonsillaris.)

2. The irritation of the rare, acute inflammation of the mucous membrane of the larynx, (laryngitis) and lower down the trachea, (cynanche trachealis,) or croup of children. I never saw but one example of the spasm from this last cause, and of this no note was taken. The child was suffocated before bronchotomy could be performed.

3. The irritation of an irritable bronchocele.
4. From the irritation of an ulcer in the œsophagus.
5. The mere handling and leeching an irritable bronchocele.
6. The irritation of a scrophulous abscess of the pharynx.
7. The irritation of a phlegmonous abscess of the neck.
8. Irritation of a chronic abscess of the pharynx.
9. Of acute phlegmonous abscess of the fauces.
10. Chronic enlargement of the tonsils, when inflamed and increased in size by taking cold, as in sore throats.
11. Venereal ulcer of the throat.
12. Elongation of the uvula.

It is probable that the degree and duration of the spasm of the glottis, in many instances, depend upon the quality of the primary irritation, and the seat of its application, more especially extraordinary sensibility of surface, which itself may be influenced by the condition of the stomach. Thus some substances taken into this organ at one period will produce head-ach, whilst at others they will *not*." 19.

The treatment of spasmodic stricture of the glottis consists in the speedy removal of its cause; if this be not effected with decision and rapidity, bronchotomy becomes necessary. In general, the duration of the spasm is short; but, if there be regular difficulty of breathing independent of it, as from affections of the larynx, &c. the danger of suffocation is greatly increased. In all doubtful cases, the most careful survey of the interior and exterior parts of the throat should be made with the hand and eye. Nearly the whole of the pharynx may be explored with the finger, if the jaws are well fixed asunder by a proper speculum; the fore-finger of the right hand introduced between the molares, as far back as the corner of the mouth on its right side will admit of, may command the whole cavity. The examination should be quickly done. Laryngitis is discovered by its symptoms; its treatment should be active in the commencent, and bold in the extreme.

In the fatal case of bronchocele, there was rather irritability than inflammation, irritability which, as our readers well know, is not to be relieved by the means adapted to inflammation. On the diagnosis we need not insist here. If the spasmodic stricture of the glottis is excited by acute inflammation of the fauces, the most active local and general means should be employed; if suppuration has taken place, we require a fair and broad opening into the abscess. Very powerful general means, and free scarification of the inflamed part will often prevent suppuration, and to do so should be our object. If an abscess, however, does form in the tonsil, accompanied by increasing dyspnœa, no time should be lost, but a careful examination should be instantly resorted to.

The abscess generally bulges through the velum, on the side on which the tonsil is most affected. The finger usually distinguishes an obscure fluctuation, but if a pharyngotomos be plunged about at random, the abscess may not be opened. An abscess of the tonsil will sometimes take place on the back part, skirting it in a long and somewhat indistinct form, like the gum abscess seen between the cheek and the jaw.

"The best instrument for opening the abscess of the tonsil is a broad lancet concealed in a sheath, called the pharyngotomos. With the aid of a speculum, which furnishes a good view, and a steady hand, it may be used effectively and safely; but there is mischief to be apprehended from carelessness, as well as from gross ignorance, even in this very simple operation: it would be the former, were a surgeon to point his instrument towards the angle of the jaw, and plunge it deep into the carotid, because he would not be at the trouble of gaining a good view: it would be something else, of an infinitely worse character, should he do this in ignorance of the situation of that vessel. And yet it has been struck more than *once* or *twice*, and the unfortunate patient sent to his account without warning by the very hand that should have saved him. Of course the pharyngotomos should be held in a straight line from one of the lateral incisors opposite the abscess, to the back of the pharynx, and striking the abscess only when the operator is certain that the mouth of the canula is resting fairly upon the spot he wishes to tap.

Should the abscess be deep in the pharynx, as in the case of Hopton and the hospital patient, a curved instrument must be used, the fore-finger of the operator leading the point of the canula to the abscess, as far towards the back of the pharynx by the spine as possible, thus acting behind the larynx. The prominence of the tumour will assist." 23.

When acute inflammation supervenes upon chronic enlargement of the tonsil, the most vigorous means should be speedily adopted. The occurrence of the spasm during sleep is probably referrible to the position of the patient and her relaxed condition, the tonsils falling cumbrously backward, and encroaching on the pharynx. When the tonsil becomes so large as seriously to interfere with respiration and deglutition, it becomes necessary to remove a large portion of it. The removal of the whole by the knife is dangerous, and patients frequently dislike the use of the knife at all. Mr. Fletcher has been in the habit of using the ligature with perfect success.

"Cheselden used to pass a curved needle, armed with a double ligature, through the base of the enlarged tonsil, by which the part was divided into two portions. The threads were then separated, and the two belonging to the upper portion were passed through a canula, (Levret,) which was carried close to the tumor, and retained there by twisting the ligatures around its bars. The other half was then treated in the same manner, and the threads on each tightened daily. This is a difficult operation to perform, especially where the jaws are not fixed by a dilator, and Mr. Chevalier somewhat improved it, in making a hole through the tonsil by means of a broad curved needle fixed in a long handle, and through that the double ligature was passed, appended to a bent probe, in the original manner of Cheselden. But even this method is sufficiently difficult and tedious. The orifice of the hole through the gland cannot always be easily discovered by the head of the probe, and then you have to tighten each thread separately. The mode I have adopted is one which renders all inconvenience to the operator, from the irritability of the fauces, impossible.

This is done by first separating the jaws of the patient by means of a dilator, so that when the fauces are teazed by the fingers of the surgeon, the first shall not be bitten, nor the latter interrupted in his operation by the patient shutting the mouth.

Before the dilator is fixed, a packer's noose, made of small whip-cord, should be prepared. This is effected by making a single knot upon one end of the thread; this end, with the knot, is to be brought forward upon the other, so as to make a single noose upon itself, including the other, and to be drawn tight upon it, close to the first knot. The free end of the thread is then to be passed through the ring of the simple instrument used by Mr. Chevalier, to tighten the knot. See his plate, 3d Vol. Med. and Chir. Transactions. So far, I go with Mr. Chevalier.

The dilator is now fixed; the jaws are well open. The assistant seizes the tonsil with a double hook, on which hangs the noose. This last, with the ring appended to it, is held by the surgeon himself. Now the assistant must pull the tonsil from its loose bed in a diagonal direction across the mouth: its base becomes elongated, the noose is slipped over it, and carried by the fingers of the surgeon close around it: the ring is then run up to the gland, and the ligature tightened, which is repeated daily till the tonsil drops off, which it does in a few days.

It may be supposed that this simple mode of noosing a tonsil could not be carried into effect where the base of the gland is very wide. But it is surprising how forcibly pulling out this part from its bed, will contract the base of it by elongation. Now and then it may so happen that the base would be too broad for the noose; I can only say, however, that I have never yet met with a tonsil with a base that would not yield to elongation, and, therefore, that could not be noosed in the way described.

If the surgeon be bold, and dexterous with his fingers, the whole operation is done with a rapidity and effect very different to the long and worrying mode pointed out by Cheselden, or the ingenious improvement of Mr. Chevalier." 25.

This concludes the chapter on Spasm of the Glottis. The cases are instructive—the remarks sensible. We have been careful to give Mr. Fletcher's opinions and experience, rather than to indulge in critical remarks. Mr. F. is surgeon to a county hospital, he publishes some of the results of his experience, and the public would rather have them than an ocean of commentary. Practical readers will form their own conclusions. We pass to the chapter on strictures of the œsophagus.

### III. On Strictures of the Œsophagus and the Dangers of the Bougie.

Mr. Fletcher reprobates the mischiefs which are produced by surgeons in their mechanical treatment of strictures of the œsophagus; he believes that false passages are made in one-half of the cases treated. To improve the methodus medendi, by diminishing its danger, is the object of the present article, and with this view he proposes a new instrument, executed by Mr. Weiss.

Setting aside the spasmodic affections, there are two kinds of firm stricture of the œsophagus. The first appears to be a contraction or puckering of the inner membrane, with but little thickening in its neighbourhood, formed of a mere transverse fold of the membrane itself, leaving an aperture in the gullet, sometimes in its centre, often close by its side, with the remainder of the tube blocked up. This may be called the membranous stricture, yet it is capable, especially if thrown diagonally across the canal, of turning the point of the already bent instrument against the side of the tube itself. If a little more force than usual be applied, it passes quite through the substance of the œsophagus. The other form of stricture of the œsophagus is a dense, cartilaginous thickening of its coats, assuming a tumour-like character, and more or less surrounding the canal. This may be and has been called a scirrhous stricture, but of scirrhus it has not exactly the structure, neither has it altogether the symptoms. Our author has not seen this cartilaginous stricture conjoined with ulceration of the œsophagus. Whatever be its nature, it is the business of the surgeon to make his way through it. The cartilaginous is a very rare form of stricture, when compared with the membranous, and the latter is not common.

Case 1. *Membranous Stricture—Death from false Passage made by the Bougie.* A maiden lady, æt. 60, who had been affected for nearly 30 years with severe forms of dyspepsia, complained of uneasiness about her throat and across the lower part of the neck, with painful and difficult deglutition. On making an examination with a small bougie, Mr. F. with some difficulty got through a stricture beyond the cricoid cartilage, but not till he had turned the point considerably to the left; it seemed to grate over a rough surface. Mr. F. passed the instrument twice during a few days, and the patient left Gloucester improved. She passed to the charge of her family surgeon, who sometimes could pass the instrument and sometimes could not. A little blood was occasionally spit up, the glands in the neck swelled, she did not swallow so well, and complained of increased pain across the root of the neck, and a tight feeling as if a cord was drawn round it. In a short time suppuration was advancing in front of two lymphatic glands, and a bougie only half the size of that previously employed could not be passed without difficulty. At times she could swallow much better than at others. In the course of a month she was very much reduced, and, although the bougie could be passed now and then, she invariably complained afterwards of pain between her shoulders, in the throat and about the sternum; she could only swallow liquids; she sometimes expectorated matter with an offensive smell.

"I was requested to see her. The abscesses had burst, but were not yet made into issues. The glands behind them were hard, and appeared to strike deep into the throat. On examination with the bougie I discovered more room at the point of the stricture than could be expected under the circumstances, and enough, I should have supposed, to allow of the passage of considerable morsels of solid food. The point of the instrument gave considerable pain at the situation of the increased opening, which reminded me of the pain suffered when an urethra bougie has made a new passage from that canal. She attempted to swallow, and it appeared that some small solid morsels passed, but milk returned. Invariably, however, this act of swallowing induced coughing, and great quantities of mucus were brought up by the mingled act of retching with coughing. She said, feebly, 'I think it went down, but it comes up again with this nasty stuff.' She now too was worried by considerable difficulty of breathing, and I took my leave of her, I was sure, for the last time in life. The print will best illustrate the dissection. The stricture itself was nearly impervious, and was formed by a diagonal fold of the inner membrane across the canal of the œsophagus. There was, however, no diseased appearance near this new arrangement of the inner membrane forming the obstruction; but a considerable opening made by the bougie was evident, connecting the œsophagus and wind-pipe. The mucous membrane of the latter was greatly and extensively inflamed, and its channel filled with mucus, among which were one or two very small substances, looking like pieces of food of some sort, that had been forced in this direction through the opening or false passage. There were no other diseased appearances likely to shorten life." 30.

The foregoing case is intended to illustrate the extreme dangers of the pointed bougie used in the ordinary manner. The following is given with another view.

### CASE 2. *Firm Cartilaginous Stricture.*

Mr. W. æt. 60, a spirit drinker first felt some difficulty in swallowing his food about eight months before his application to Mr. Fletcher. His voice was then hoarse and husky, and great portion of his food was rejected, but he was otherwise in good health. In the course of a month after this Mr. F. passed a bougie, the size of his little finger, through a stricture, with such difficulty as to convey an impression of its being unusually firm and resisting.

Gradually the difficulty of passing instruments increased, the point being turned back by the firmness of the stricture, whilst the difficulty of swallowing increased to such a degree, that he could only swallow a little port wine. He was supported in some degree by injections of strong broth, yet he seemed to be sinking fast, when, after various trials with other means, our author curved a large urethra metallic bougie to the shape of the fauces, and, having measured the distance of the stricture, succeeded in passing the obstruction. By moving the instrument laterally with some freedom, more room was obtained, and the patient swallowed almost immediately. For a week he swallowed meat and did well, but the patient caught cold, and on the eighth day the bougie would not pass, the patient was feverish, his throat was sore, and the fauces swollen. The larynx now sympathised, and he breathed laboriously. Leeches were applied, he became weak, and although a tube was introduced into the œsophagus and egg and wine thrown into the stomach, spasm of the glottis supervened and he died in the night.

On examination the stricture of the œsophagus was apparently of scirrhous character, a section discovering a hard whitish, cartilaginous thickening, occupying a considerable portion of the circle of the œsophagus. There was no ulceration near it nor any where in the canal, nor did the cut surface of the thickening give the appearance of the membranous septa common to scirrhus. Above the stricture the œsophagus had a rugous character, strongly contrasted with its smoothness below it.

"In the first of these cases, it is sufficiently clear that the patient's life was shortened by the making of a false passage with the bougie, into the windpipe. It is probable that a false passage thus made must always prove fatal; for, besides the difficulty of knowing when an instrument might be clear of it, and in the natural track of the œsophagus, there would also be another danger in the delay of waiting for the healing of this new opening, before attempts could again be made to find the natural channel; a delay that, in an enfeebled patient, would probably be ruinous.

The stricture must be daily and rapidly closing, the chance of success constantly diminishing, as there would be no power, even in occasional dilatation, to delay for a short time the fatal narrowing of the tube. In the urethra the case is different. The stream of urine, though diminished in size, passing frequently along its canal, would probably assist in checking its final closure from a stricture, when an interruption to the use of the bougie became necessary, from a false passage having been made by this instrument. This stream too, coming in a contrary direction from that of the false passage, passes over its small orifice without entering it. But such advantages do not exist, where a false passage has been made from the œsophagus; no mechanical power for a moment keeps open the stricture, for none can safely reach it, from the danger of its taking the new road. There is no natural dilating power from the direction of the stomach, which would be the safest road. The only one left is so full of danger, that no prudent traveller would venture to encounter its perils. The bougie would follow the track of the new and ruinous opening it had already made, and food itself, the only natural means of temporary dilatation, could not be safely employed, for in the patient's attempt at swallowing, it would enter the trachea, (should the opening be in that direction,) and there excite a degree of inflammation in its mucous lining, that would greatly assist in bringing about a fatal termination, as in the case of Miss B. already described.

A false passage, therefore, from the œsophagus, is a much more important accident than one from the urethra; so teeming with mischief, and disappointment in the treatment of this interesting though desperate case of real stricture of the œsophagus, that too much pains cannot be taken in the formation of instruments, or in their employment, to avoid such a mishap.

In the female patient, the stricture was strictly membranous. There cannot be the least suspicion of malignancy in the nature of this case, and therefore I feel no hesitation in believing, that her life would have been saved by the destruction of the stricture. This might certainly have been effected by an instrument which could pass the stricture safely, and afterwards by its construction, and in its retreat, possess the power of breaking asunder the folds of membrane constituting it.

In a case like the second, there may be doubts about the complete success of any instrument, but still in this, the one described would be superior to all others, inasmuch that it may be used with perfect safety, as far as the danger of making a false passage is concerned. It may be objected, that in the case of a scirrhous obstruction of the œsophagus, that the part would not dilate, and that the disease might be exasperated by the use of the instrument presently to be described. But it does not always happen, that there are no dilatable points left in the circumference of the œsophagus. The scirrhous thickening may only occupy one side or point of the tube, and in regard to the exasperation of the scirrhus into cancer, we must first have evidence of the existence of the former state, which is not to be obtained, unless in one particular case, where the surgeon could not be mistaken.

If the patient has an ulcerated scirrhus (cancer) of the œsophagus, he will spit pus mixed with blood, there will be pain of a lancinating nature about his throat—his pulse will quicken—his look be cadaverous—and he will more quickly emaciate than can be accounted for by the diminished quantity of food he swallows. In such a case, the chance of success from any instrument would indeed be but small, and the friends of the patient should know that nothing more than relief could be expected here. But, would a surgeon be justified even then in retiring from the patient; in doing nothing, in letting the tube be closed by the disease, without making an effort to widen it for the passage of food—for the prolongation of life—when he has full probability that it is in his power to carry this measure into effect?

In the worst possible case therefore of the stricture of the œsophagus, I am decidedly of opinion that a surgeon would not do his duty, who did not attempt to remove it, at least so as to clear a road to the stomach. Life is always dear to the majority of mankind, and a prolongation of it is frequently desirable. In the foregoing case of cartilaginous stricture, there cannot be a doubt of its prolongation, and even a strong probability of an ultimate cure being effected, had an instrument, such as I have the honour of submitting to the profession, been employed. The coarse and uncertain movements of the metallic sound çà et là, in improving the passage, were so decided and remarkable, as to induce the patient and his friends to believe that a cure was certain. How much more efficient would an instrument be, whose operation must have a precision, a power of dilating more or less, or even of lacerating, according to the will of the operator?" 35.

The instruments now employed are the bougie made of cloth and wax; the probang, with an ivory ball and whalebone handle; the elastic gum bougie; the caustic bougie. To each and all of these Mr. F. urges objections, and many, if not most, are no doubt well founded, but we need not enumerate them here. Mr. F. has twice known the common bougie pushed through the œsophagus, and that by good surgeons. He has also once seen the elastic gum bougie driven through the lower part of the pharynx into the neck amongst the great vessels, around which it excited suppuration.

Case 3. *Fatal false Passage made by the Bougie through the Pharynx.*

The patient had stricture of the œsophagus, impassable to a bougie, immediately opposite the cricoid cartilage, which had been inflamed by armed bougies to such an extent as not to permit the smallest quantity of solid food, and very little liquid to pass the obstruction. A surgeon thought that he had succeeded in passing a stiffened elastic gum bougie with a sharp point. On the following day the throat was enormously swollen, a large abscess followed, and from the combined effects of starvation and the irritation of the abscess he died. A plate shews the perforation of the pharynx in the spot previously mentioned.

"The instrument I have projected, as a substitute for pointed ones, may be used as a lacerator of the stricture, or as a mere dilator of it. It acts from its sides. It is made of metal, curved to the shape of the throat. The size of it is small, so as to allow its point to pass through any stricture with certainty, and without the slightest force. I have never met with a stricture of the œsophagus in life or in death, that would not readily permit the passage of so small an instrument.

The stricture which destroyed the patient, the subject of the second case, admitted a metallic sound of twice the size of the dilator, although it was impassable to solid food. The drawing will best explain the structure of the dilator for the œsophagus stricture. A ball of steel is at the point of the instrument when it is closed; by turning its handle, this ball separates the instrument into three divisions, ascends in the centre of them, and in its route, enlarges the size of the dilator, as may be required either to dilate or to destroy. Its use should be prefaced by an accurate examination of the seat of the stricture, which is usually behind the cartilages of the larynx, and most commonly below the cricoid. The head of the patient being thrown well back, and the jaws fixed asunder by the mouth dilator, a middle sized brown bougie, curved and made soft in hot water, should be passed down the pharynx to the stricture, which is generally distant from the edge of the dentes incisores, about six or seven inches, or according to the stature of the patient, or length of the neck. The stricture struck, its distance should be marked by the thumb-nail on the instrument, as it lies under the teeth. The soft point of the bougie will have received some impression from the stricture, and you may judge from this, and from the slight force you have employed, what sized dilator may be required. Having selected the proper size, the distance of the stricture from the teeth may be marked upon it with a small dentist, or Lancashire file, or any other mark; the exact admeasurement being copied from the soft bougie employed in the examination. The ball of the closed dilator should now be placed against the back of the pharynx, down which it should be permitted to slide till it reaches the stricture, or when it is ascertained that the mark is immediately below the edge of the teeth. If it should pass the stricture readily, but perceptibly, for an inch and a half, or till the file-mark be over the tongue, it has gone far enough, and the operator taking the instrument in his left hand, near to its handle, should turn this last with his right, and its point will now be expanded, according to the width that may be required. It is now to be slowly, and steadily withdrawn through the stricture, which in its retrograde passage, it either tears or dilates, according to the judgment of the operator, from his knowledge of the quality or narrowness of the obstruction. If he wishes to dilate only, he will open the dilator slightly in the first instance, and, should the patient not then be enabled to swallow on its being withdrawn, he will repeat the introduction, and increase the dilatation until sufficient room be obtained—to admit of the passage of food to the stomach." 39.

A lithographic plate of the instrument is given, but we think the description will render its nature sufficiently intelligible. When the stricture has been sufficiently opened for the safe introduction of the instrument it may be used as a mere dilator. Should the patient be suffering from inanition and the dilatation slow, a hollow tube may be easily passed through the stricture, and liquid food injected into the stomach by Weiss's syringe. The quantity given should not exceed three ounces at a time, which may be repeated several times a day. Overloading the stomach should be most scrupulously avoided. Our author dwells on the mechanical treatment of stricture of the œsophagus, because he conceives that if this is to be cured it is by downright surgery and nothing else. Physic is useless, and may be given to the dogs, with as much utility as to the patient. With the following sentiments he concludes, and so do we.

"It is now thirty-two years since I entered the wards of the Gloucester Infirmary, as a pupil. It is a large hospital. But such is the rarity of real or permanent obstructions of the gullet, that in hospital and private practice combined, I cannot satisfactorily make out a list of more than fourteen cases, which occurred during the whole of that long period. The majority of these were treated medically—they perished. Others were treated with the pointed bougie, simple, or armed—I fear this treatment was equally unsuccessful. Where opportunities occurred, in these last cases, of inspection after death, false passages, or openings through the substance of the tube, were generally discovered, which either killed the patient outright, or accelerated his fate.

Preserve me, therefore, from the bougie which is to dilate or force this kind of stricture with its point!

Let its use be ever so skilfully conducted—the surgeon ever so remarkable for his knowledge—coolness—delicacy—decision, and manual dexterity, rare combination indeed! still would I not suffer him to approach my person, had I a stricture of the œsophagus, armed with a bougie to act from its point. To escape from so dangerous an individual, would be the first and rational impulse of the mind, for man clings to life instinctively, even when he knows it to be burthensome." 42.

Some other portions of Mr. Fletcher's work remain to be noticed, which they will be on proper occasions. From the space we have devoted to its consideration, our readers very readily conceive that we entertain a favourable opinion of it; indeed we wish that all surgeons or physicians of experience would follow our eloquent author's example. Unfortunately the majority pursue a plan diametrically opposite; with them the books come first, the experience, if at all, afterwards. When we meet with a writer of the former class we welcome him with cordiality. If we have a fault to find with Mr. F. it is touching his style. For medical writing it is somewhat too fine, too rhetorical. But this is a trfling blemish, and we part from Mr. Fletcher with feelings of so much consideration and respect, that we experience great pleasure in the expectation of meeting him again.

